# Association between warfarin use and thromboembolic events in patients post-Fontan operation: propensity-score overlap weighting analyses

**DOI:** 10.1093/ejcts/ezae413

**Published:** 2024-11-19

**Authors:** Wakana Maki, Shotaro Aso, Ryo Inuzuka, Hiroki Matsui, Kiyohide Fushimi, Hideo Yasunaga

**Affiliations:** Department of Clinical Epidemiology and Health Economics, School of Public Health, The University of Tokyo, Tokyo, Japan; Department of Real-world Evidence, Graduate School of Medicine, The University of Tokyo, Tokyo, Japan; Department of Pediatrics, The University of Tokyo, Tokyo, Japan; Department of Health Services Research, Graduate School of Medicine, The University of Tokyo, Tokyo, Japan; Department of Health Policy and Informatics, Tokyo Medical and Dental University Graduate School of Medical and Dental Sciences, Tokyo, Japan; Department of Clinical Epidemiology and Health Economics, School of Public Health, The University of Tokyo, Tokyo, Japan

**Keywords:** Fontan procedure, Thrombosis, Bleeding events, Thromboprophylaxis, Warfarin, Observational study

## Abstract

**OBJECTIVES:**

The appropriate antithrombotic regimen after a Fontan operation is yet to be elucidated. Hence, this study aimed to compare the incidence of thromboembolic events in patients with and without receiving warfarin for thromboprophylaxis in a large post-Fontan population.

**METHODS:**

This retrospective cohort study used data from the Diagnosis Procedure Combination database in Japan between April 2011 and March 2022. We identified all patients who underwent a Fontan operation and excluded those who were born before 2010, died during the hospitalization or received mechanical heart replacement. Propensity score overlap weighting was performed between patients discharged with warfarin (with or without aspirin) and the control group (only aspirin or neither aspirin nor warfarin). Cox and Fine-Gray hazards models compared thromboembolic and bleeding events.

**RESULTS:**

We identified 2007 eligible patients, including 1670 warfarin users and 337 non-users. The mean follow-up duration was 2.1 years. The crude proportions of thromboembolic events were 3.0% and 3.0% and those of bleeding events were 0.4% and 0.3% in the warfarin and control groups, respectively. There was no significant difference in thromboembolic events between the groups (sub-distribution hazard ratio: 0.77; 95% confidence interval 0.39–1.51; *P* = 0.45) or bleeding events (sub-distribution hazard ratio: 0.78; 95% confidence interval 0.09–7.03; *P* = 0.83).

**CONCLUSIONS:**

Warfarin use at discharge after a Fontan operation may not be necessary for thromboembolism prophylaxis in paediatric patients, based on large-scale real-world data, with a mean postoperative follow-up duration of 2.1 years. There is room for further studies to reconsider routine warfarin use in patients post-Fontan operation.

## Graphical Abstract

**Figure:**
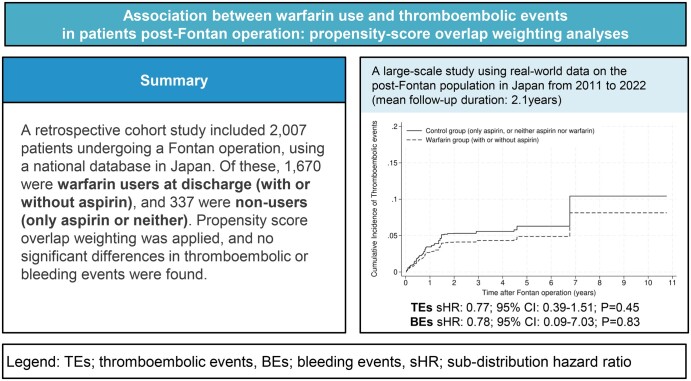

## INTRODUCTION

Fontan operation is the culmination of palliative heart operation for individuals with a single functional ventricle. However, patients who received a Fontan operation face an increased risk of thrombosis [1–4]. Thromboembolic events exhibit a bimodal distribution, with an incidence ranging from 1% to 33%. The 1st peak occurs within 6 months after the operation, followed by another peak 10 years later [2, 4, 5]. Thrombosis causes morbidity and mortality in patients with a Fontan operation [4, 6]; therefore, it is of utmost importance to prevent thromboembolic events for such patients.

The most appropriate antithrombotic regimen in patients with a Fontan operation is yet to be elucidated [2]. In several clinical guidelines, aspirin and warfarin are recommended or considered reasonable for 3–12 months after a Fontan operation; moreover, long-term therapy is also considered reasonable, especially for patients with thromboembolic risk factors. However, the evidence levels of each recommendation are low [7–9]. Consequently, in actual clinical practice, thromboprophylaxis in this population depends on the clinicians’ preference and varies widely [2, 10].

Thromboprophylaxis, including aspirin and/or warfarin, has demonstrated greater efficacy than no therapy [11, 12]. However, these previous studies were heterogeneous because of a lack of consensus in defining the exposure of thromboprophylaxis and the outcome of thrombotic events. The indications for thromboprophylaxis were not clear, and confounding factors were not adjusted. One prior study compared warfarin and aspirin using propensity score matching to adjust for patient characteristics; however, the sample size was small (*n* = 475) [13].

The aim of the present study was to compare the incidence of thromboembolic events in patients with and without receiving warfarin for thromboprophylaxis in a large post-Fontan population. We focused on warfarin as thromboprophylaxis after the Fontan operation because warfarin is difficult to manage in children. We hypothesized that the administration of warfarin would be associated with a lower incidence of thrombotic events without an increased risk of bleeding, aligning with previous studies.

## MATERIALS AND METHODS

### Ethical statement

This study was performed in line with the principles of the Declaration of Helsinki. Written informed consent was not required due to the anonymity of patients in the database. This study was approved by the Institutional Review Board of the University of Tokyo [approval number: 3501-(5); 19 May 2021].

### Data source

In this retrospective cohort study, we used the Diagnosis Procedure Combination database, a national database of inpatients in acute-care institutions in Japan. The details of this database have been described previously. Approximately 1200 institutions in Japan, including 82 academic institutions, are included in the database and provide data on ∼8 million inpatient admissions annually, representing ∼50% of all the acute-care inpatients in Japan [14]. A previous validation study reported that the sensitivity and specificity of the recorded primary diagnoses were 78.9% and 93.2%, respectively, while those of recorded procedures exceeded 90% [15].

The database included the following information: age; sex; diagnoses recorded with text data in Japanese and according to the International Classification of Diseases, Tenth Revision (ICD-10) codes (including main diagnosis, comorbidities present at admission, and conditions that arose after admission); daily records of procedures including surgical operations; medication; length of stay; and discharge status.

### Study participants

We identified all patients who received a Fontan operation from 1 April 2011 to 31 March 2022 in the database, as this timespan represents the maximum period covered by our database. We excluded patients born before 2010 because we considered a Fontan operation to usually be conducted after the age of 12 months. We also made this decision because we were not able to distinguish whether a particular Fontan operation involved reoperation if patients underwent the 1st Fontan operation prior to April 2011. In a prior study in Japan, the median age at the time of the Fontan procedure was 3 years, with a minimum age of 1 year, further supporting our age-exclusion criterion [16]. We also excluded patients who died prior to discharge. We also excluded those who received mechanical heart valve replacement before or at the time of Fontan operation because these patients were strongly advised to be on warfarin [7–9, 13].

### Exposure

The exposure variable was the prescription of warfarin at discharge after a Fontan operation. In our main analysis, the patients were divided into the warfarin and control groups. We did not consider prescription changes at any point following discharge, as the database we used did not contain any information on outpatient prescriptions. In this primary analysis, we treated aspirin prescription as a covariate. Additionally, we compared 4 groups, based on the prescriptions of warfarin and aspirin at discharge following the Fontan operation, as follows: patients who received neither warfarin nor aspirin [W(–) A(–) group], patients who received only warfarin [W(+) A(–) group], patients who received only aspirin [W(–) A(+) group] and patients who received both warfarin and aspirin [W(+) A(+) group]. Because this grouping was based solely on discharge prescriptions, there was no possibility of patients switching groups.

### Outcomes

The primary outcome was post-Fontan thromboembolic events requiring hospitalization. Based on previous studies, we identified the following thromboembolic events from the database: retinal vascular occlusion (ICD-10 codes: H34), acute coronary syndrome (I24.9), acute myocardial infarction (I21), intracardiac thrombosis (I51.3), pulmonary embolism (I26), arterial thromboembolism (I74), transient ischaemic attack (G45), cerebral infarction (I63), renal embolism or thrombosis (N28.0), splenic infarction (D73.5), lower extremity phlebitis (I80), hepatic vein thrombosis or phlebitis of portal vein (I81, K76.5, K75.1), embolism and thrombosis of other veins (I82), intracranial phlebitis and thrombophlebitis (G08), and embolism of prosthetic devices, implants and grafts (T82.8) [17–20]. We were not able to identify the correct onset date of thromboembolic events. Therefore, we treated the admission date of the concerned hospitalization caused by thromboembolic events as the onset date. We only observed the 1st thromboembolic event and treated it as a censoring event because our investigation focused on the association between warfarin prescription at discharge (which was assumed to be continued after discharge) and the 1st event after discharge.

The secondary outcome was bleeding events, which required any surgical interventions. We identified the following bleeding events from the database: intracranial bleeding (I61), intraspinal bleeding (G95.1), pericardial haematoma (I31.2), intra-abdominal haematoma or retroperitoneal haematoma (K66.1), intra-articular bleeding (M25.0), intraocular bleeding (H44.8), compartment syndrome (M62.2), gastrointestinal bleeding (K92.0–K92.2) and respiratory haemorrhage (R04.2 and R04.8) during hospitalizations [17, 21–23]. The surgical interventions to treat bleeding involved vascular embolization, endoscopic gastrointestinal haemostasis and endoscopic oesophageal and gastric variceal ligation. We were not able to differentiate major bleeding events from non-major ones because our database did not contain information on volume of blood loss.

### Covariates

The covariates included: sex, original anatomical diagnosis, types of pre-Fontan heart operations, age at Fontan operation, calendar year of Fontan operation, pre-Fontan home oxygen therapy, medications, thrombotic events, bleeding events, postoperative use of extracorporeal membrane oxygenation, Fontan reoperations, presences of prosthetic conduits and patches, presence of pacemakers or implantable cardioverter defibrillators and comorbidities including atrial arrhythmia [2, 6, 17, 24] (Supplementary Material, Table S1). In the main analysis, aspirin prescription at discharge was treated as a covariate. In the matching weight analysis in which we compared 4 groups based on warfarin and aspirin prescription, we treated aspirin prescription as exposure. The included original anatomical diagnoses and their ICD-10 codes are shown in Supplementary Material, Table S1. Types of pre-Fontan heart operations included prior pulmonary artery banding, prior aortopulmonary shunt and prior staging with bidirectional cavopulmonary shunt. Pre-Fontan medications included diuretics, angiotensin-converting enzyme inhibitors, angiotensin receptor blockers, β-blockers and antiarrhythmic drugs (detailed in Supplementary Material, Table S1). Pre-Fontan thrombotic and bleeding events were the same as those used in the outcome definitions. The included comorbidities and their ICD-10 codes are also presented in Supplementary Material, Table S1 [24]. All comorbidities were diagnosed before the Fontan operation. We did not include prescription of direct oral anticoagulants as a covariate because it was considered uncommon in our population.

We did not exclude patients with prior thrombotic and/or bleeding events, and we treated the previous thrombotic/or bleeding events as one of the potential risk factors [17].

### Statistical analyses

Continuous variables are expressed as medians and interquartile ranges, and categorical variables are expressed as numbers and percentages. Inter-group differences in outcomes were analysed using the chi-squared test for categorical variables.

We conducted propensity score overlap weighting analyses. Furthermore, we performed multivariable logistic regression analysis to estimate the propensity scores for receiving warfarin at discharge. We used the covariates as possible confounders. A generalized estimation equation was fitted to the regression model to adjust for in-hospital clustering. The C-statistic was used to discriminate between the models. Each patient was weighted by the predicted probability of receiving the opposite treatment. Standard differences were calculated to assess the balance of covariates between the 2 groups, and an absolute mean difference of more than 10% indicated imbalance. The details of the overlap weighting analysis methods have been described previously [25].

A Cox regression model was used to compare differences between the weighted warfarin and control groups. A Fine-Gray model was also used to obtain a sub-distribution hazard ratio (SHR), in which we considered death by other causes or heart transplant as a competing risk [26]. We defined death by other causes as when a patient died in hospital and did not have the diagnosis of thromboembolic and/or bleeding disease. The day of the 1st Fontan operation was defined as the index date. We followed the patients through the 1st thromboembolic event, death, heart transplant or the end of follow-up (31 March 2022), whichever happened 1st.

Subgroup stratifications were conducted based on the diagnosis of atrial arrhythmia, history of thrombosis and aspirin prescription at discharge. To determine whether the treatment effects differed significantly across various subgroups, we 1st calculated the interaction *P*-value. In the subgroup analyses, the same propensity score as that used in the main analysis was applied, and the SHRs of thromboembolic events were measured as well as the main analysis.

Matching weight analysis was conducted to compare the outcome of thromboembolic events among the 4 groups. These groups were based on the prescriptions of warfarin and aspirin at discharge after the patient’s Fontan operation: patients who received neither warfarin nor aspirin [W(–) A(–) group], patients who received only warfarin [W(+) A(–) group], patients who received only aspirin [W(–) A(+) group] and patients who received both warfarin and aspirin [W(+) A(+) group]. We set the W(–) A(–) group as the control group. Matching weight analysis, an extension of propensity score analysis, is used for comparison among 3 or more treatment groups and demonstrates improved matching [27].

We conducted 6 sensitivity analyses. First, we limited the patients who underwent only 1 Fontan operation because the risk of thromboembolic events changes after Fontan reoperation. Second, the index date was shifted to 1 year after the operation, considering the possibility of changes in prescription details during outpatient visits after discharge. In this analysis, we included only the patients who were admitted to the hospital within 1 year of a Fontan operation and whose follow-up period was longer than 1-year post-Fontan. Because we were not able to obtain data on outpatient visits, the patients who received warfarin at every discharge within 1 year of their Fontan operations were sorted into the warfarin group and the others into the control. Third, we excluded patients prescribed warfarin preoperatively, as well as those with a preoperative history of thromboembolic or bleeding events, as these histories were considered strong eligibility criteria for either prescribing or not prescribing warfarin postoperatively. Fourth, we included all the patients irrespective of their birth year, including those born before 2010, who were excluded from the primary analysis. Fifth, we limited the follow-up period to a maximum of 1 year to focus on early-onset outcomes. Sixth, we redefined bleeding events as the recorded diagnosis of bleeding events, regardless of the requirement of the surgical intervention.

No patients had any missing data for any of the included covariates. All statistical analyses were performed using Stata software (version 17.0; StataCorp LP, College Station, TX) and the threshold for significance was set at a *P*-value of <0.05.

## RESULTS

**Figure 1:**
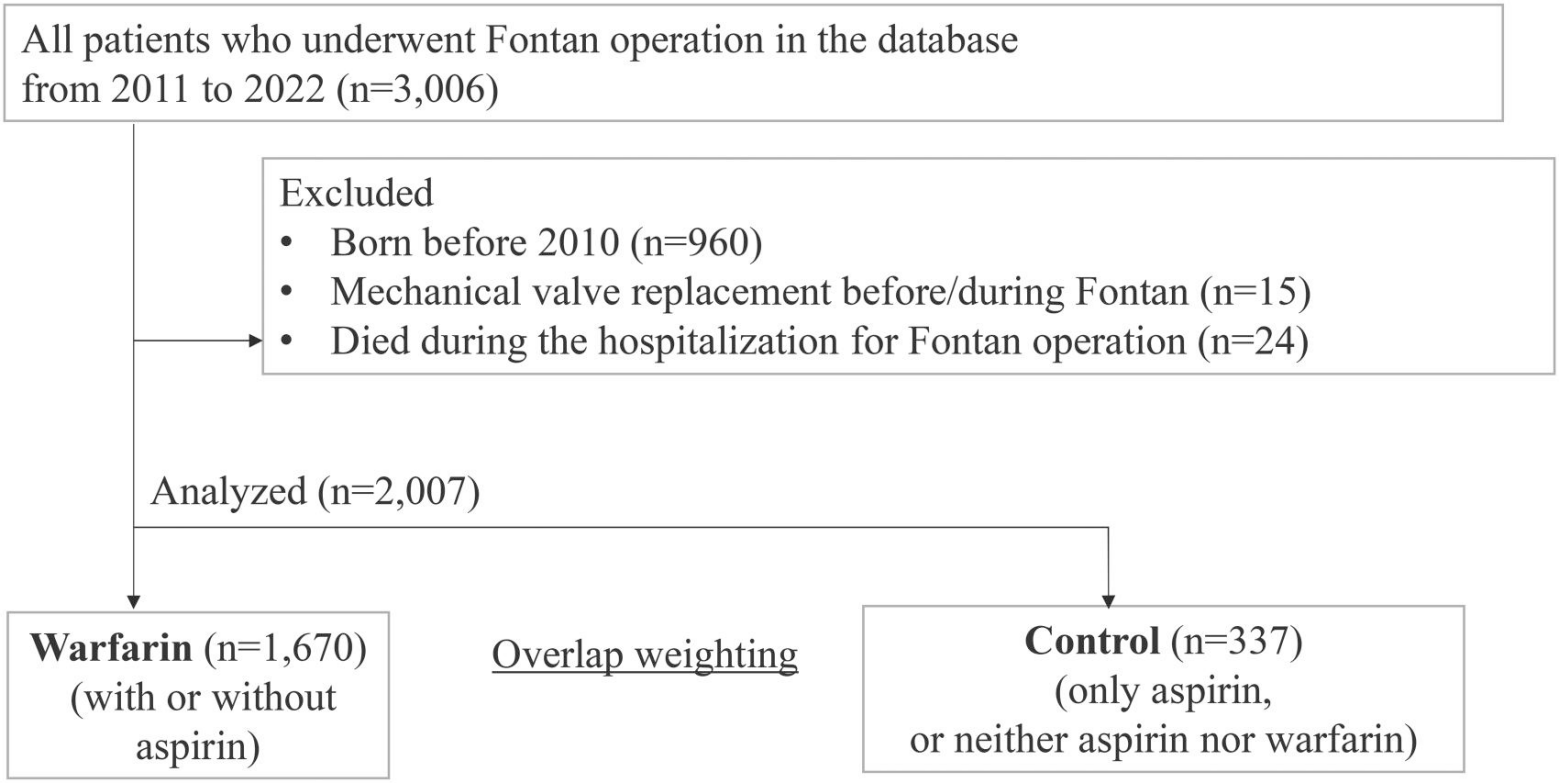
Flow chart for participant selection.

Figure 1 shows the flow chart of patient selection. We identified 3006 patients who underwent a Fontan operation during the study period. After applying the exclusion criteria, we identified 2007 patients eligible for our study, including 1670 (83.2%) in the warfarin group and 337 (16.8%) in the control. The mean follow-up duration was 2.1 years.

Table 1 shows the baseline characteristics of the 2 groups before and after overlap weighting analysis. Patients in the warfarin group were more likely to: be older at Fontan, have major aortopulmonary collateral arteries, have received thiazides and carvedilol, have a history of thrombosis before Fontan and have received aspirin at discharge. Patients in the warfarin group were less likely to: have transposition of great arteries and mitral atresia; have undergone pulmonary artery banding and surgeries using arterial patches; have received loop diuretics, angiotensin-converting enzyme inhibitors, and other β blockers than bisoprolol and carvedilol; and have a history of bleeding events.

**Table 1:** Demographic and clinical characteristics of the patients in the warfarin and control (no warfarin) groups

	All patients			Overlap-weighted patients		
	Warfarin (*N* = 1670)	Control (*N* = 337)	ASD (%)	Warfarin	Control	ASD (%)
Male, *n* (%)	957 (57.3)	190 (56.4)	1.9	(55.8)	(55.8)	0.0
Original anatomical diagnosis, *n* (%)						
Heterotaxia	308 (18.4)	50 (14.8)	9.7	(16.0)	(15.9)	0.2
Tricuspid atresia	260 (15.6)	56 (16.6)	2.9	(16.4)	(16.4)	0.0
Hypoplastic left heart syndrome (HLHS)	302 (18.1)	65 (19.3)	3.1	(18.7)	(18.7)	0.1
Pulmonary atresia with intact ventricular septum (PAIVS)	461 (27.6)	82 (24.3)	7.5	(26.1)	(26.0)	0.2
Atrioventricular septal defect (AVSD)	300 (18.0)	54 (16.0)	5.2	(16.9)	(16.8)	0.2
Transposition of great arteries (TGA)	165 (9.9)	46 (13.6)	11.7	(12.6)	(12.6)	0.1
Congenitally corrected transposition of great arteries (CCTGA)	68 (4.1)	14 (4.2)	0.4	(4.0)	(4.0)	0.0
Double-outlet right ventricle (DORV)	447 (26.8)	95 (28.2)	3.2	(28.3)	(28.3)	0.0
Mitral atresia (MA)	92 (5.5)	31 (9.2)	14.2	(8.3)	(8.3)	0.1
Ebstein disease	40 (2.4)	6 (1.8)	4.3	(2.0)	(2.0)	0.0
Ventricular septal defect without other diagnoses	22 (1.3)	9 (2.7)	9.7	(2.2)	(2.2)	0.0
Types of pre-Fontan heart operations						
Pulmonary artery banding (PAB)	451 (27.0)	110 (32.6)	12.3	(29.9)	(29.9)	0.1
Aortopulmonary shunt	581 (34.8)	123 (36.5)	3.6	(37.1)	(37.0)	0.2
Bidirectional cavopulmonary shunt (BCPS)	1237 (74.1)	257 (76.3)	5.1	(75.5)	(75.4)	0.1
Age at Fontan operation, mean (standard deviation)	2.7 (1.2)	2.4 (0.9)	32.0	2.4(0.9)	2.4(0.9)	0.2
Calendar year of Fontan operation, *n* (%)						
2011	13 (0.8)	6 (1.8)	8.9	(1.4)	(1.4)	0.0
2012	84 (5.0)	36 (10.7)	21.1	(8.7)	(8.7)	0.1
2013	146 (8.7)	61 (18.1)	27.7	(14.4)	(14.3)	0.2
2014	189 (11.3)	46 (13.6)	7.1	(13.8)	(13.9)	0.1
2015	175 (10.5)	44 (13.1)	8.0	(13.0)	(13.0)	0.1
2016	208 (12.5)	39 (11.6)	2.7	(12.4)	(12.4)	0.1
2017	204 (12.2)	28 (8.3)	12.9	(9.5)	(9.5)	0.1
2018	170 (10.2)	15 (4.5)	22.1	(5.4)	(5.4)	0.1
2019	154 (9.2)	13 (3.9)	21.8	(4.8)	(4.8)	0.1
2020	176 (10.5)	16 (4.7)	21.9	(5.9)	(5.9)	0.1
2021	131 (7.8)	29 (8.6)	2.8	(9.5)	(9.4)	0.1
2022	20 (1.2)	4 (1.2)	0.1	(1.1)	(1.1)	0.0
Pre-Fontan home oxygen therapy, *n* (%)	1002 (60.0)	205 (60.8)	1.7	(61.0)	(61.1)	0.1
Pre-Fontan medications, *n* (%)						
Loop diuretics	1546 (92.6)	326 (96.7)	18.6	(96.1)	(96.1)	0.0
Thiazides	253 (15.1)	31 (9.2)	18.3	(10.7)	(10.7)	0.0
Angiotensin-converting enzyme (ACE) inhibitors	1038 (62.2)	257 (76.3)	30.9	(72.8)	(72.8)	0.1
Angiotensin receptor blockers (ARB)	46 (2.8)	12 (3.6)	4.6	(3.4)	(3.4)	0.0
Bisoprolol	39 (2.3)	8 (2.4)	0.3	(2.4)	(2.4)	0.0
Carvedilol	273 (16.3)	41 (12.2)	12.0	(13.3)	(13.3)	0.0
Other beta blockers	157 (9.4)	45 (13.4)	12.5	(12.2)	(12.3)	0.0
Amiodarone	45 (2.7)	11 (3.3)	3.3	(3.2)	(3.2)	0.0
Sotalol	15 (0.9)	5 (1.5)	5.4	(1.3)	(1.3)	0.0
Pre-Fontan thrombotic events, *n* (%)	313 (18.7)	46 (13.6)	13.8	(14.1)	(14.1)	0.1
Pre-Fontan bleeding events, *n* (%)	3 (0.2)	4 (1.4)	12.2	(0.6)	(0.6)	0.0
Postoperative use of extracorporeal membrane oxygenation, *n* (%)	60 (3.6)	8 (2.4)	7.2	(2.7)	(2.7)	0.0
Fontan reoperations, *n* (%)	14 (0.8)	2 (0.6)	2.9	(0.7)	(0.7)	0.0
Presences of prosthetic conduits, *n* (%)	1654 (99.0)	332 (98.5)	4.8	(98.7)	(98.7)	0.0
Presences of prosthetic patches, *n* (%)	1357 (81.3)	309 (91.7)	30.8	(90.5)	(90.4)	0.1
Presence of pacemakers or implantable cardioverter defibrillators, *n* (%)	21 (1.3)	1 (0.3)	11.0	(0.4)	(0.4)	0.1
Pre-Fontan Comorbidities, *n* (%)						
Major aortopulmonary collateral arteries (MAPCA)	464 (27.8)	61 (18.1)	23.2	(20.1)	(20.0)	0.2
High blood pressure	295 (17.7)	46 (13.6)	11.1	(14.9)	(14.9)	0.1
Heart failure	1331 (79.7)	272 (80.7)	2.5	(80.8)	(80.7)	0.1
Diabetes	10 (0.6)	4 (1.2)	6.2	(0.7)	(0.7)	0.0
Coagulation abnormalities	24 (1.4)	5 (1.5)	0.4	(1.6)	(1.5)	1.0
Atrial arrhythmia	134 (8.0)	29 (8.6)	6.9	(8.3)	(8.3)	0.1
Polycythaemia	2 (0.1)	0 (0.0)	4.9	(0.2)	(0.0)	6.3
Abnormal liver function	4 (0.2)	0 (0.0)	6.9	(0.1)	(0.0)	2.1
Abnormal renal function	18 (1.1)	5 (1.5)	3.6	(0.2)	(0.2)	0.0
Protein losing enteropathy	1 (0.1)	0 (0.0)	3.5	(0.0)	(0.0)	0.3
Aspirin prescription at discharge	1204 (72.1)	223 (66.2)	12.8	(67.7)	(67.7)	0.0

ASD: absolute standard difference.

The overall incidence of post-Fontan thromboembolic events was 3.0% (62/2007), 1.5 per 100 patient-years, and the overall incidence of post-Fontan bleeding events was 0.4% (8/2007), 0.14 per 100 patient-years (detailed in Supplementary Material, Table S2). Before weighting, the incidence of thromboembolic events was not significantly lower in the warfarin group than the control (3.0% vs 3.0%, respectively; *P* = 0.58). Furthermore, the incidence of bleeding events was not significantly higher in the warfarin group than the control (0.4% vs 0.3%, respectively; *P* = 0.74). The overall mortality was 0.5% (11/2007), which was not statistically significant between the warfarin and control groups (0.5% vs 0.6%, respectively; *P* = 0.90).

**Figure 2:**
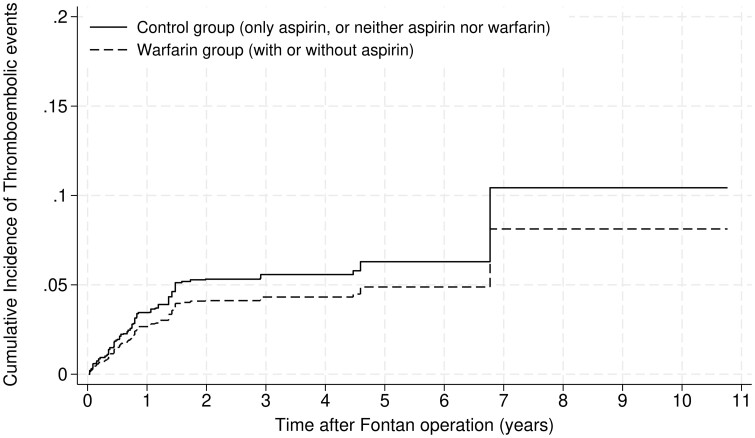
Cumulative incidences of thromboembolic events requiring hospitalization in the overlap-weighted warfarin and control (non-warfarin) groups.

**Figure 3:**
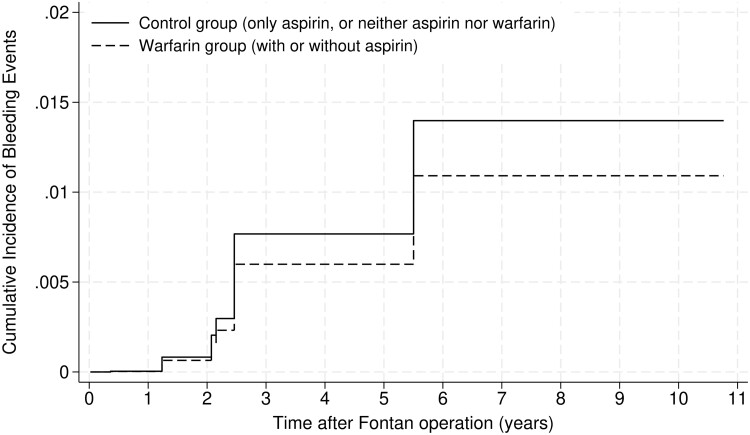
Cumulative incidences of bleeding events requiring hospitalization and any surgical procedures in the overlap-weighted warfarin and control (non-warfarin) groups.

In the overlap weighting analysis, all the variables were well-balanced (Table 1), and the C-statistic was 0.75. Table 2 shows the results of the SHR of the warfarin group compared with the control. There were no significant differences between the warfarin and control groups in the incidence of thromboembolic events [SHR: 0.77; 95% confidence interval (CI) 0.39–1.51; *P* = 0.45; effective sample size = 978]. There were also no significant differences between the warfarin and control groups in the incidence of bleeding events (SHR: 0.78; 95% CI 0.09–7.03; *P* = 0.83). The cumulative incidences of thromboembolic and bleeding events in the overlap-weighted patients are presented in Figures 2 and 3, respectively.

**Table 2:** Propensity score-overlap weighted sub-distribution hazard ratios (SHRs) in the warfarin group compared with the control (no warfarin) group among patients undergoing a Fontan operation

	SHR	95% Confidence interval	*P*-value	Interaction *P* value
Thromboembolic events requiring hospitalization				
Primary analysis (All patients born in and after 2010)	0.77	0.39, 1.51	0.45	–
Subgroup analysis				
Atrial arrhythmia				0.06
With	3.34	0.42, 26.71	0.25	–
Without	0.62	0.31, 1.28	0.20	–
History of thromboembolic events before Fontan				0.01
With	0.53	0.19, 1.49	0.23	–
Without	0.95	0.39, 2.31	0.91	–
Aspirin prescription at discharge	–	–	–	0.32
With	0.63	0.26, 1.57	0.33	–
Without	0.97	0.35, 2.72	0.96	–
Sensitivity analysis				
Among patients with only 1 Fontan operation	0.77	0.39, 1.51	0.44	–
Shifting the index date to the 1 year after the Fontan	0.36	0.10, 1.35	0.13	–
Among patients without warfarin prescription or a history of thrombotic or bleeding events before the Fontan	1.21	0.43, 3.44	0.72	–
Including patients who were born before 2011	0.86	0.50, 1.48	0.60	–
Bleeding events requiring hospitalization and any surgical interventions				
Primary analysis (all patients born in and after 2010)	0.78	0.09, 7.03	0.83	
Bleeding events requiring hospitalization				
Primary analysis (all patients born in and after 2010)	0.21	0.05, 0.80	0.02	–

In the subgroup analyses, the interaction *P*-values were 0.06, 0.01 and 0.32 for a diagnosis of atrial arrhythmia, history of thromboembolic events and aspirin prescription at discharge, respectively. Prescription of warfarin at discharge was not associated with the incidence of thromboembolic events, irrespective of a history of thromboembolic events (*n* = 359; SHR: 0.53; 95% CI 0.19–1.49; *P* = 0.23) (Table 2). Although the interaction *P*-values exceeded 0.05, a prescription of warfarin at discharge was not associated with the incidence of thromboembolic events, irrespective of the diagnosis of atrial arrhythmia (*n* = 163; SHR: 3.34; 95% CI 0.42–26.71; *P* = 0.25) or aspirin prescription at discharge (*n* = 1427; SHR: 0.63; 95% CI 0.26–1.57; *P* = 0.33).

In the matching weight analysis, the patients were distributed with 5.7% (114/2007) in the W(–) A(–) group, 23.2% (466/2007) in the W(+) A(–) group, 11.1% (223/2007) in the W(–) A(+) group and 60.0% (1204/2007) in the W(+) A(+) group. Of these, the 1670 patients in the warfarin group in the main analysis included the 466 patients in the W(+) A(–) group and the 1204 patients in the W(+) A(+) group. After the matching weight analysis, almost all the covariates were well-balanced (Table 3). Compared to the W(–) A(–) group, the other 3 groups did not have a significantly lower risk of thromboembolic events [SHR of the W(+) A(–) group: 0.96; 95% CI 0.31–3.00, SHR of the W(–) A(+) group: 0.62; 95% CI 0.15–2.51, SHR of the W(+) A(+) group: 0.47; 95% CI 0.16–1.41; effective sample size  = 590].

**Table 3:** Matching weighted sub-distribution hazard ratios (SHRs) of thromboembolic events in the warfarin (+) aspirin (–) group, the warfarin (–) aspirin (+) group, the warfarin (+) aspirin (+) group compared with the control [warfarin (–) aspirin (–)] group among patients undergoing a Fontan operation

Prescriptions after a Fontan operation	SHR	95% Confidence interval	*P*-value
Warfarin (–) Aspirin (–)	1 (Ref)	–	–
Warfarin (+) Aspirin (–)	0.96	0.31, 3.00	0.94
Warfarin (–) Aspirin (+)	0.62	0.15, 2.51	0.50
Warfarin (+) Aspirin (+)	0.47	0.16, 1.41	0.18

Table 2 shows the results of the sensitivity analyses. In patients who underwent only 1 Fontan operation, warfarin was not associated with the incidences of thromboembolic events (*n* = 1991; SHR: 0.77; 95% CI 0.39–1.51; *P* = 0.44). Moreover, when we shifted the index date to 1 year after the Fontan, warfarin was not associated with the incidences of thromboembolic events (*n* = 618; SHR: 0.36; 95% CI 0.10–1.35; *P* = 0.13). The result remained consistent after excluding patients with a preoperative prescription of warfarin, as well as those with a history of thrombotic or bleeding events before the surgery (*n* = 1083; SHR: 1.21; 95% CI 0.43–3.44; *P* = 0.72). After including patients born before 2010, warfarin was not associated with the incidences of thromboembolic events (*n* = 2919; SHR: 0.86; 95% CI 0.50–1.48; *P* = 0.58) either. When we limited the follow-up period to a maximum of 1 year, all cases of outcome events (thromboembolic events; *n* = 12 and bleeding events, which required surgical interventions; *n* = 1) were observed in the warfarin group. Finally, when we defined bleeding events as the recorded diagnosis of bleeding events, a prescription of warfarin at discharge was found to be significantly associated with a lower risk of bleeding events (SHR: 0.21; 95% CI 0.05–0.80; *P* = 0.022).

## DISCUSSION

Warfarin use after a Fontan operation was not significantly associated with a lower incidence of thromboembolic events or a higher incidence of bleeding events compared with non-warfarin use. Our findings were inconsistent with those of previous studies. Two previous meta-analyses showed that patients with some thromboprophylaxis were significantly less likely to have thromboembolic events after a Fontan operation [11, 12]. However, the indications for thromboprophylaxis in the previous studies were unclear. Therefore, high-risk patients were more likely to receive thromboprophylaxis. Moreover, the outcome definitions of thromboembolic and bleeding events varied among the studies. The present study included a larger sample size and adjusted for as many confounding factors as possible, which may have increased the comparability of the warfarin and control groups.

Although the point estimate of our main result demonstrated a lower risk of thromboembolic events in the warfarin group, the difference was not significant. Our sensitivity analyses supported the main result. Likewise, when we considered the prescription of warfarin and aspirin in the matching weight analysis, any type of thromboprophylaxis was not associated with a significant lower risk of thromboembolic events. Conversely, a prescription of warfarin and aspirin showed a relatively lower risk in the 4 groups although the difference did not reach significance; therefore, a combination of these two medications may be effective. Our follow-up duration of 2.1 years was shorter than those of previous studies with up to 7.1 and 8.1 years [11, 12]. However, our study delved into the peak time for thromboembolic events, occurring 1 year post-Fontan operation [2, 4, 5]. In fact, the incidence of thromboembolic events in our study, at 1.5 per 100 patient-years, was compatible with the range of 1.3–3.5 per 100 patient-years previously reported [12]. Although our sample size was larger than those in previous studies, the results may have been underpowered to detect a significant difference. Another possible reason for our inconsistent findings was that we only included patients born in and after 2011. Though our database did not contain the details of operations, most of our patients probably underwent a new-type Fontan operation; that is, total cavopulmonary connection (lateral tunnel or extracardiac conduit), instead of an old one, atriopulmonary connection or right atrium–right ventricle connection. Although the risk difference of thromboembolic events between the Fontan types remains known, several old reports suggested that the new Fontan type had a lower risk of thrombosis [28, 29]. Another study showed no significant difference in the risk of thrombosis between the new cavopulmonary anastomosis types (lateral tunnel or extracardiac conduit) [30].

In our sensitivity analysis, in which the follow-up period was limited to a maximum of 1 year after Fontan operation, thromboembolic and bleeding events were observed only in the warfarin group. However, we were unable to elucidate the reasons for these findings in this study due to the absence of information regarding diagnostic modalities or the symptomatic nature of the events in our database, highlighting the need for further research. Additionally, our study did not include events occurring between the Fontan procedure and discharge, as precise onset dates for these events were not available. As such, considering events prior to discharge might yield different results.

When we redefined bleeding events as the recorded diagnosis of bleeding events, irrespective of needs for any surgical procedures, warfarin was found to be significantly associated with a lower risk of bleeding events; however, we did not elucidate the underlying reasons in the current study. Although we adjusted for as many baseline characteristics as possible, including aspirin prescription, the adjustment for variables such as liver dysfunction and coagulation abnormalities was based solely on recorded diagnoses, not on laboratory data.

### Limitations

The present study had several limitations. First, the diagnoses in our database were not perfect, with a sensitivity of only 78.9%. Therefore, it is likely that some baseline characteristics and outcome events were underreported in this study [15]. Further, our findings are based on an observational study, and therefore, unobserved confounders such as reporting biases in diagnoses and procedures between the groups may exist. Second, our database did not contain information on details of Fontan operations. We did not consider the presence of fenestration and/or residual shunts, which were previously reported to be risk factors of thromboembolism [31]. We were not able to acquire intraoperative data such as circulatory arrest time, original dominant ventricle, ventricular dysfunction and valve regurgitation. No laboratory data such as international normalized ratio were available. Control of warfarin therapy was not taken into consideration though poor warfarin control is a risk factor for thromboembolism [5]. Third, we only evaluated prescriptions at discharge and comorbidities before the Fontan operation. Therefore, the prescriptions the patients received may have changed for any reason between discharge and the end of follow-up. Further, the patients may have had new-onset comorbidities, such as arrhythmia, protein-losing enteropathy and collateral arteries, which may have affected medication choice during follow-up. Because our primary focus in this study was warfarin prescription at discharge, we conducted all analyses based on an intention-to-treat principle. In addition, we conducted the sensitivity analysis shifting the index date from the Fontan operation date to 1 year after the operation to account for changes in prescriptions that occurred during the 1st postoperative year, while the results remained consistent. Fourth, we did not consider patient medication compliance. Therefore, the effectiveness of warfarin and/or aspirin may be underestimated in this study. Fifth, ∼70% of the patients were prescribed aspirin at discharge. Therefore, our findings may indicate the combined effect of aspirin and warfarin rather than the effect of warfarin alone. Sixth, we did not have the protocols for detecting thromboembolic events or their clinical severities. An international guideline recommended further surveillance imaging tests for thromboembolism in patients with risk factors post-Fontan operation, although not strongly [7]. Hence, it is possible that the subclinical thromboembolic events of the high-risk patients tended to be detected more often. Last, most of our population were Asian. A previous study suggested that Asian patients with Fontan operations would be less prothrombotic than Caucasians [30]. This may have contributed to our findings.

## CONCLUSIONS

In conclusion, our findings, based on large-scale real-world data, suggest that warfarin use after a Fontan operation may not be necessary for thromboembolism prophylaxis in paediatric patients following Fontan operation during a relatively short follow-up period, with a mean follow-up duration of 2.1 years; however, it may not increase bleeding risk either. Warfarin is a difficult medication to manage, especially in children, because it requires frequent blood monitoring. There is room for further studies to reconsider routine warfarin use in patients post-Fontan operation.

## Supplementary Material

ezae413_Supplementary_Data

## Data Availability

The authors do not have permission to share data due to reasons of sensitivity.

## ABBREVIATIONS

CI: Confidence interval
ICD-10: International Classification of Diseases, Tenth Revision
SHR: Sub-distribution hazard ratio
