# Supplementary material for: Association between warfarin use and thromboembolic events in patients post-Fontan operation: propensity-score overlap weighting analyses
Source: Eur J Cardiothorac Surg. 2024 Nov 19;66(6):ezae413. doi: 10.1093/ejcts/ezae413 (PMC11604171; doi:10.1093/ejcts/ezae413)
Supplement: ezae413_Supplementary_Data [file ezae413_supplementary_data.docx]

**Supplemental Table 1. Covariates and their definitions**

| Sex | Male or female |
| --- | --- |
| Original anatomical diagnosis | Heterotaxia (Q89.0), tricuspid atresia (Q22.4), hypoplastic left heart syndrome (HLHS) (Q23.4), pulmonary atresia with intact ventricular septum (PAIVS) (Q25.5), atrioventricular septal defect (AVSD) (Q21.2), transposition of great arteries (TGA) (Q20.3), congenitally corrected transposition of great arteries (CCTGA) (Q20.5), double outlet right ventricle (DORV) (Q20.1), mitral atresia (MA) (Q23.2), Ebstein disease (Q22.5), and ventricular septal defect (Q21.0), without other diagnoses listed above using the ICD-10 codes |
| Types of pre-Fontan heart operations | pulmonary artery banding (PAB), aortopulmonary shunt, and bidirectional cavopulmonary shunt (BCPS) |
| Age at Fontan operation | In days |
| Calendar year of Fontan operation | 2011 to 2022 |
| Pre-Fontan conditions |  |
| -home oxygen therapy | Yes or No |
| -medications | Diuretics (loop diuretics and thiazides), angiotensin-converting enzyme (ACE) inhibitors, angiotensin receptor blockers (ARB), β-blockers (bisoprolol, carvedilol and others), and antiarrhythmic drugs (amiodarone and sotalol) |
| -Thrombotic events requiring hospitalization | retinal vascular occlusion (ICD-10 codes: H34), acute coronary syndrome (I24.9), acute myocardial infarction (I21), intracardiac thrombosis (I51.3), pulmonary embolism (I26), arterial thromboembolism (I74), transient ischemic attack (G45), cerebral infarction (I63), renal embolism or thrombosis (N28.0), splenic infarction (D73.5), lower extremity phlebitis (I80), hepatic vein thrombosis or phlebitis of portal vein (I81, K76.5, K75.1), embolism and thrombosis of other veins (I82), intracranial phlebitis and thrombophlebitis (G08), and embolism of prosthetic devices, implants, and grafts (T82.8) |
| -Bleeding events requiring hospitalization and any surgical interventions | Intracranial bleeding (I61), intraspinal bleeding (G95.1), pericardial hematoma (I31.2), intra-abdominal hematoma or retroperitoneal hematoma (K66.1), intra-articular bleeding (M25.0), intraocular bleeding (H44.8), compartment syndrome (M62.2), gastrointestinal bleeding (K92.0–K92.2), and respiratory hemorrhage (R04.2 and R04.8) during hospitalizations |
| Postoperative use of extracorporeal membrane oxygenation | Yes or No |
| Fontan reoperations | Yes or No |
| Presences of prosthetic conduits and patches | Yes or No |
| Presence of pacemakers or implantable cardioverter defibrillators | Yes or No |
| comorbidities | Major aortopulmonary collateral arteries (MAPCA) (Q25.8), high blood pressure (I10–15), heart failure (I50), diabetes (E10–14), coagulation abnormalities (D66, D67, D68.0–D68.2, D68.5), atrial arrhythmia (I47.1, I48), polycythemia (D75.1), abnormal liver function (K70–77), abnormal renal function (N18, N19, K28.9, R94.4), and protein-losing enteropathy (K90.4) |
| Aspirin prescription at discharge | Not used in the matching weight analysis |

**Supplemental Table 2. Crude incidences of thromboembolic and bleeding events requiring surgical interventions in the warfarin and control (no warfarin) groups**

|  | Warfarin  (N=1,670) | Control  (N=337) |
| --- | --- | --- |
| **Thromboembolic events*** |  |  |
| Acute coronary syndrome | 1 | 0 |
| Intracardiac thrombosis | 1 | 0 |
| Arterial thromboembolism | 30 | 6 |
| Transient ischemic attack | 4 | 2 |
| Lower extremity phlebitis | 8 | 3 |
| Embolism and thrombosis of other veins | 7 | 0 |
| Embolism of prosthetic devices, implants, and grafts | 0 | 1 |
| **Bleeding events requiring surgical interventions** |  |  |
| Gastrointestinal bleeding | 4 | 1 |
| Respiratory hemorrhage | 3 | 0 |

* The total number of thromboembolic events is 63 instead of 62, because one patient in the warfarin group experienced multiple events simultaneously.
